# Comparative Analysis of Intravitreal Diffusion Patterns Across Ex Vivo Human and In Vivo/Ex Vivo Animal Models

**DOI:** 10.1167/iovs.67.5.56

**Published:** 2026-05-20

**Authors:** Anfisa Ayalon, Avigail Beryozkin, Katherine A. Davoli, Tong Yu, Jiantao Pu, Kira L. Lathrop, Jose-Alain Sahel, Joseph N. Martel, Leah Byrne

**Affiliations:** 1 Department of Ophthalmology, School of Medicine, University of Pittsburgh, Pittsburgh, Pennsylvania, United States; 2 Department of Radiology, University of Pittsburgh, Pittsburgh, Pennsylvania, United States; 3 Department of Bioengineering, University of Pittsburgh, Pittsburgh, Pennsylvania, United States; 4 Department of Ophthalmology, University of Pittsburgh Medical Center, Pittsburgh, Pennsylvania, United States

**Keywords:** fluorescein dyes, human eye, molecular weights, pig eye, vitreous barrier

## Abstract

**Purpose:**

To investigate vitreous barrier function by examining ocular distribution patterns of anionic fluorescein dextran molecules with varying molecular weights (MWs) following intravitreal injection and to assess the influence of injection site and plasmin pretreatment on dye distribution.

**Methods:**

We studied dye distribution across five MWs (3 kDa, 40 kDa, 70 kDa, 500 kDa, and 2 MDa) in an ex vivo pig eye model, with temporal or nasal injections. For experiments involving ex vivo pig eyes pretreated with plasmin, in vivo pig eyes, and ex vivo human eyes, we utilized only 40-kDa and 2-MDa dyes injected temporally.

**Results:**

No significant differences were observed between nasal and temporal injections. In ex vivo pig eyes, 3-kDa fluorescein showed near-complete vitreous diffusion within 24 hours. The 40-kDa and 70-kDa dyes continued to spread beyond 24 hours, whereas the 500-kDa and 2-MDa dyes demonstrated restricted distribution with minimal change up to 48 hours. In vivo pig eyes showed limited distribution of 2-MDa fluorescein. Plasmin pretreatment did not affect 2-MDa distribution but enhanced the spread of 40-kDa fluorescein. In ex vivo human eyes, 40-kDa fluorescein distributed widely, whereas 2-MDa fluorescein remained highly localized and delineated a vitreous bursa structure that the dye did not penetrate.

**Conclusions:**

We observed a size-dependent relationship between dye molecules and their spread throughout the vitreous cavity, highlighting the role of vitreous as a barrier that can influence the distribution and effectiveness of treatments, including gene therapies. Future research should focus on improving the mobilization of larger substances within the vitreous while recognizing its potential as a natural reservoir for slow drug release when injected into the vitreous bursae.

The vitreous is a transparent, gel-like structure that makes up most of the volume of the eye.^1^ Although the vitreous is predominantly composed of water, it contains additional components that contribute to its complexity.^1^^–^^4^ Given that the vitreous is a key site for drug delivery in treating posterior segment pathologies, understanding its barrier function in substance distribution upon intravitreal injection is crucial, especially when developing new intraocular drugs and enhancing the efficiency of existing ones. Several studies have investigated the impact of the vitreous barrier on gene therapy and the development of new intravitreal delivery systems.^3^^,^^4^ Peeters et al.^3^ examined the distribution of fluorescein isothiocyanate (FITC)–dextrans, polystyrene nanospheres, and DNA/cationic liposome complexes (LPXs) in a portion of the extracted bovine vitreous.^3^ They demonstrated that FITC–dextrans with molecular weights (MWs) of 167 kDa, 464 kD, and 2 MDa did not significantly bind to vitreous strands and could freely move within the vitreous network, resulting in homogeneous spread. These authors suggested that hydrophobic interactions may play a significant role in immobilizing polystyrene nanospheres and LPXs because coating them with hydrophilic polyethylene glycol (PEG) increased mobility.^3^ Xu et al.^4^ also studied the spread of polystyrene nanoparticles (NPs) on the cut bovine eye with different charges, noting that positively charged NPs were immobilized but negatively charged NPs showed better spread. They further investigated the distribution of different-sized PEG-coated (neutral charge) NPs and concluded that the average mesh size of bovine vitreous is 550 ± 50 nm.^4^ These previous studies on the vitreous barrier function have indicated limitations in the spread within the vitreous of hydrophobic substances, positively charged molecules, and large molecules. Still, most of these studies worked in the bovine vitreous. The distribution of drugs was studied after vitreous separation from the underlying intraocular structures or after cutting the eye, which may not fully reflect the behavior observed in the whole intact eye, in the human eye, or in other animal models, whether ex vivo or in vivo.^3^^,^^4^ Our study aimed to investigate the entire vitreous barrier function by examining the distribution of negatively charged fluorescein dextran dyes with varying MWs and sizes following intravitreal injection across intact eyes of three models: ex vivo and in vivo pig eye models, as well as an ex vivo human eye model. Additionally, in the ex vivo pig model experiments, we subgrouped injections into temporal and nasal sites to assess potential differences. We also evaluated fluorescein distribution across time, at 24 and 48 hours post-injection, and we studied changes in fluorescein distribution in eyes after pharmacological vitreolysis.

## Methods

To study the function of the vitreous barrier, we designed experiments that utilized the entire globe by simulating intravitreal injections performed in the ophthalmology clinic. We injected 50 µL of dye in each experiment because this volume is commonly used for intraocular injections in clinical practice, as it typically does not cause a significant increase in intraocular pressure.^5^ Among various potential animal models, we selected the pig because its eyes exhibit similarities to human eye size and mechanical properties of the vitreous, and they are commonly used in research.^6^^–^^8^ FITC–dextrans with an anionic charge of varying MWs ranging from 3 kDa to 2 MDa were sourced from the same supplier, and we specifically opted for negatively charged FITC–dextrans to eliminate potential electrostatic interactions, as described by Xu et al.^4^

### Fluorescein-Only Injection in Ex Vivo Pig Eyes

#### Animals

All ex vivo fresh pig eyes were sourced from the meat production industry and were utilized within 6 hours of extraction. The parameters of the pigs included the following: Yorkshire, Duroc, or Hampshire breeds; live weight of 275 to 325 pounds (125–147 kg); 6 to 9 months old; and eye diameters of 19.93514 to 25.62603 mm. Eye diameters were recorded for all samples (Supplementary Table S1). Eyes were cleaned of periocular tissue and examined for external damage, such as cuts in the sclera, a cloudy cornea, or bleeding in the anterior chamber. Only eyes with no visible damage were included in the study.

#### Dyes

We used anionic Invitrogen FITC–dextran dyes (Thermo Fisher Scientific, Waltham, MA, USA) with five different MWs: 3 kDa, 40 kDa, 70 kDa, 500 kDa, and 2 MDa. It is important to note that the MWs of the dyes correspond to their molecular size: Dyes with higher MWs indicate larger molecular sizes (Table).^9^ All dyes were reconstituted in a standard saline solution to a concentration of 10 mg/mL as recommended by the manufacturer and vortexed until complete solubility of the conjugates was achieved. Each eye used in the experiment was injected with 50 µL of dye.

**Table.** Stokes Radii for FITC–Dextran Molecular Weights^9^

MW	Stokes Radius (nm)
2 MDa	27
500 kDa	14.7
70 kDa	5.8
40 kDa	4.5

#### Tissue Holding and Injection Method

**Figure 1.**
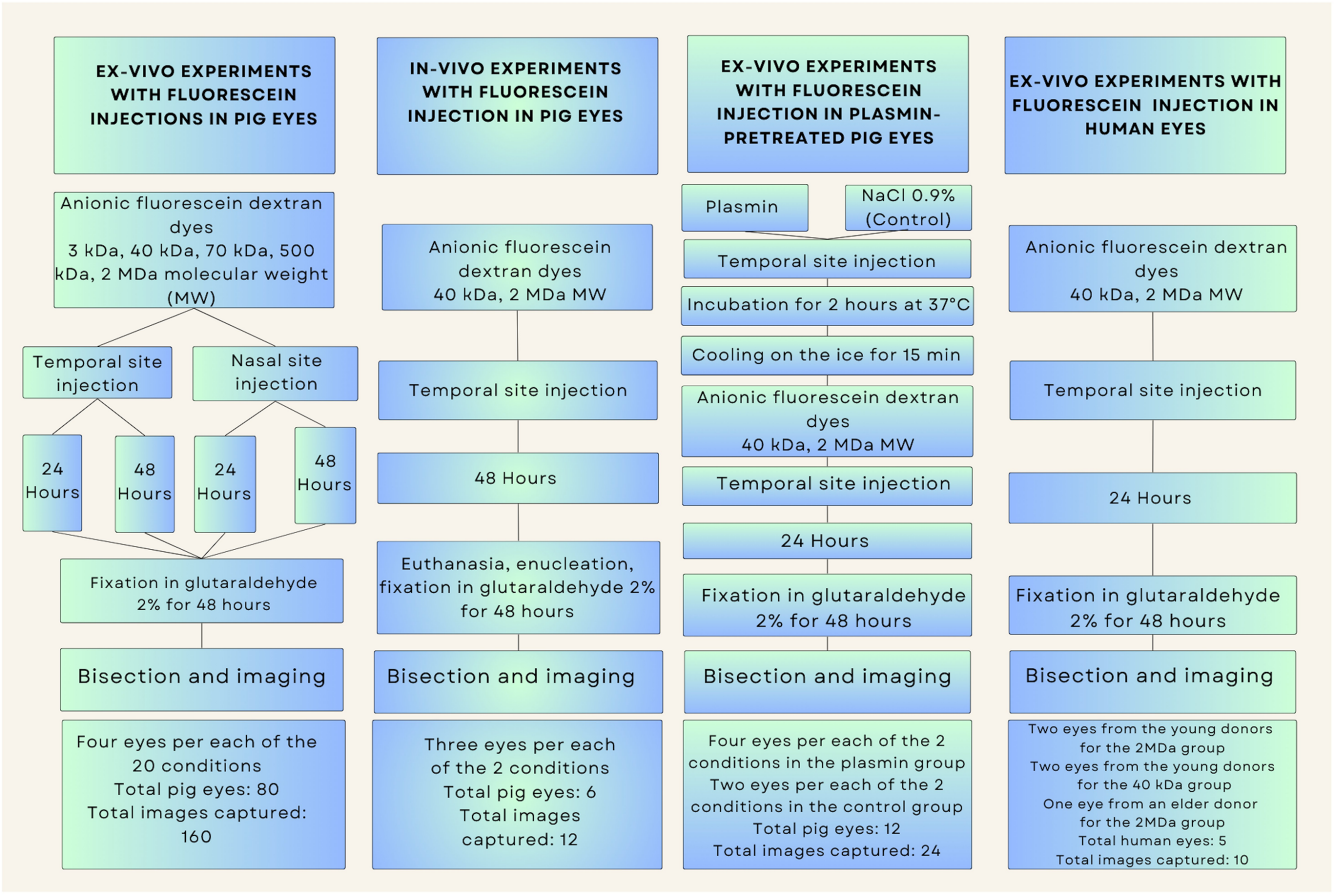
Schematic presentation of the four types of experiments detailed in the Methods section.

**Figure 2.**
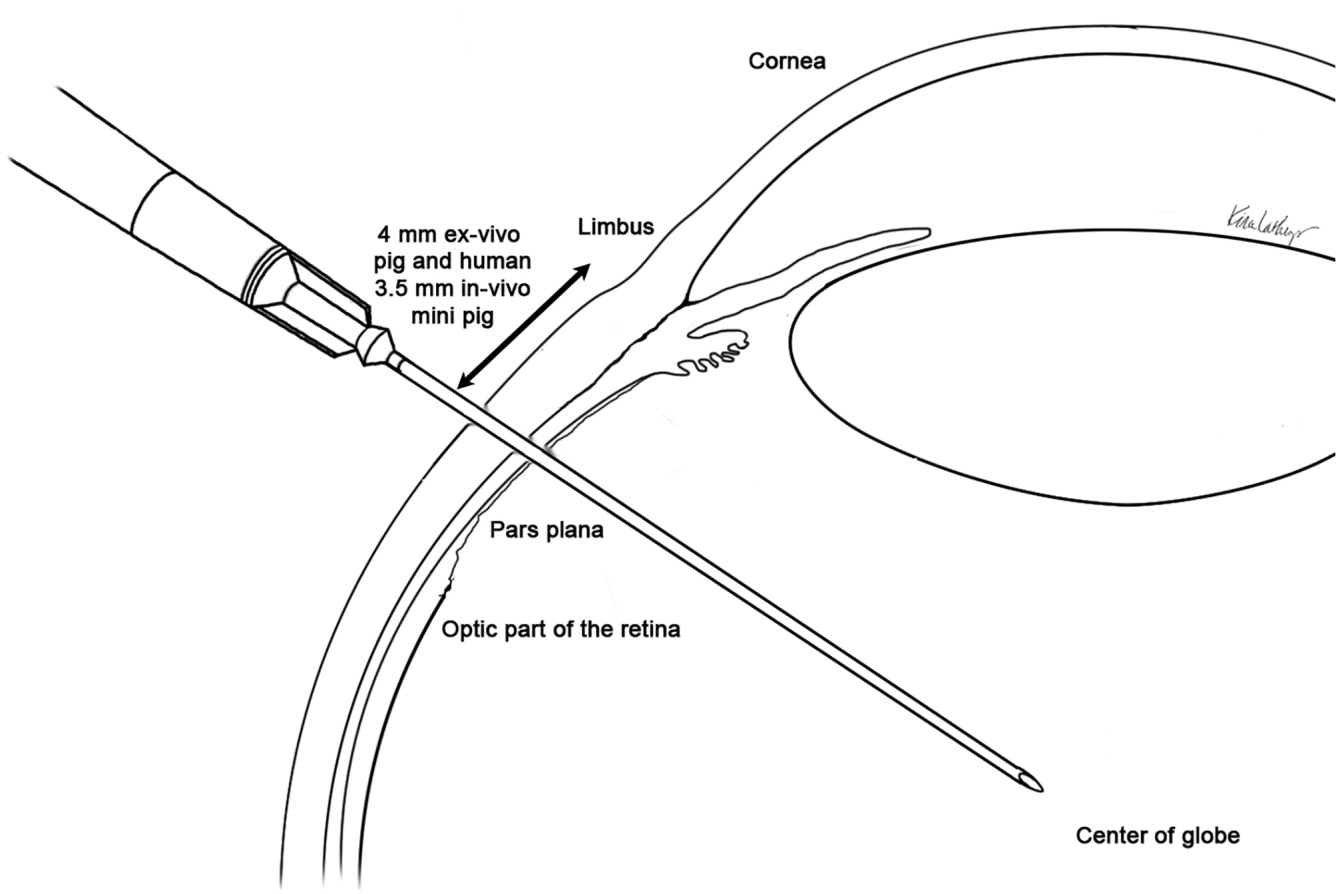
Schematic illustration of a pars plana intravitreal injection technique used in the experimental settings. The needle was introduced through the pars plana at approximately 3.5 to 4.0 mm posterior to the limbus (3.5 mm in vivo in the miniature pig eyes and 4.0 mm ex vivo in the pig and human eyes) and directed toward the center of the globe, avoiding the lens and retina. Key anatomical landmarks including the cornea, limbus, pars plana, and optic retina are depicted.

The structure of the experiments is shown in Figure 1. The experimental samples were treated as follows: We performed either nasal or temporal injections to investigate the potential impact of the injection site on dye distribution for each MW. A 30-gauge 1/2-inch needle was employed to inject 50 µL of dye pars plana (Fig. 2). The injection site was 4 mm from the limbus, as determined using surgical calipers and markings. Following scleral penetration, the needle was advanced toward the center of the vitreous cavity, the solution was gently injected, and the needle was then carefully removed. Eyes were incubated in 1× PBS (14190250; Thermo Fisher Scientific) at 4°C for either 24 or 48 hours post-injection, with corneas oriented upward in conical tubes on a slow-speed shaker. These two time points were selected to evaluate whether dye distribution patterns were time dependent. The 48-hour maximum duration minimized tissue degradation, and gentle agitation simulated physiological eye movements to facilitate dye spread. Corneal orientation was standardized to ensure balanced dye distribution between temporal and nasal regions.

#### Eye Fixation Procedure and Bisection Technique

After they were shaken, the eyes were removed, and a corneal incision was made to facilitate penetration of fixative into the eye. They were placed into conical tubes filled with freshly made vitreous fixative comprised of 2% glutaraldehyde (diluted from 25% ampoules; 16221; Electron Microscopy Sciences, Hatfield, PA, USA) in 0.1-M sodium cacodylate buffer (diluted from 0.2 M; 11652; Electron Microscopy Sciences) and left to fix for 48 hours at room temperature, with the cornea pointing up. After 48 hours of fixation, the eyes were rinsed with 1× PBS and further grossed and imaged.

Eyes were bisected in a coronal plane using a disposable high-profile microtome blade (DT554S50; Sturkey, Lebanon, PA, USA) in a single guillotine-like cutting motion to minimize damage and tissue traction during the procedure. The cut was made 10 mm posterior to the limbus, dividing the eye into two portions: One part included the anterior segment of the eye and some of the central portion of the fixed vitreous. In contrast, the second part mainly included central and posterior fixed vitreous along with the posterior pole of the eye. The anterior and posterior parts were imaged separately.

#### Photography and Picture Analysis

Images were captured using a widefield microscope (SZX16; Olympus, Tokyo, Japan) in multicolor and fluorescein modes. In total, 160 images from 80 ex vivo pig eyes were captured and analyzed using a combination of in-house software, a MATLAB-based image analysis tool (MathWorks, Natick, MA, USA), an artificial intelligence–based online program (Biodock; https://www.biodock.ai/), and ImageJ (National Institutes of Health, Bethesda, MD, USA).

Raw images were acquired under identical microscope settings. Background fluorescence was subtracted using signal measured in green fluorescent protein (GFP)-negative regions prior to normalization. Images were then normalized using linear intensity rescaling to an 8-bit grayscale range (0–255), in which the minimum and maximum pixel intensities of each image were mapped to 0 and 255, respectively. This procedure was applied uniformly across all samples to standardize image dynamic range, reduce variability arising from differences in image acquisition conditions, and enable consistent threshold-based segmentation. Normalization preserved the relative spatial distribution of fluorescence within each image and did not affect the comparative trends observed between experimental groups.

**Figure 3.**
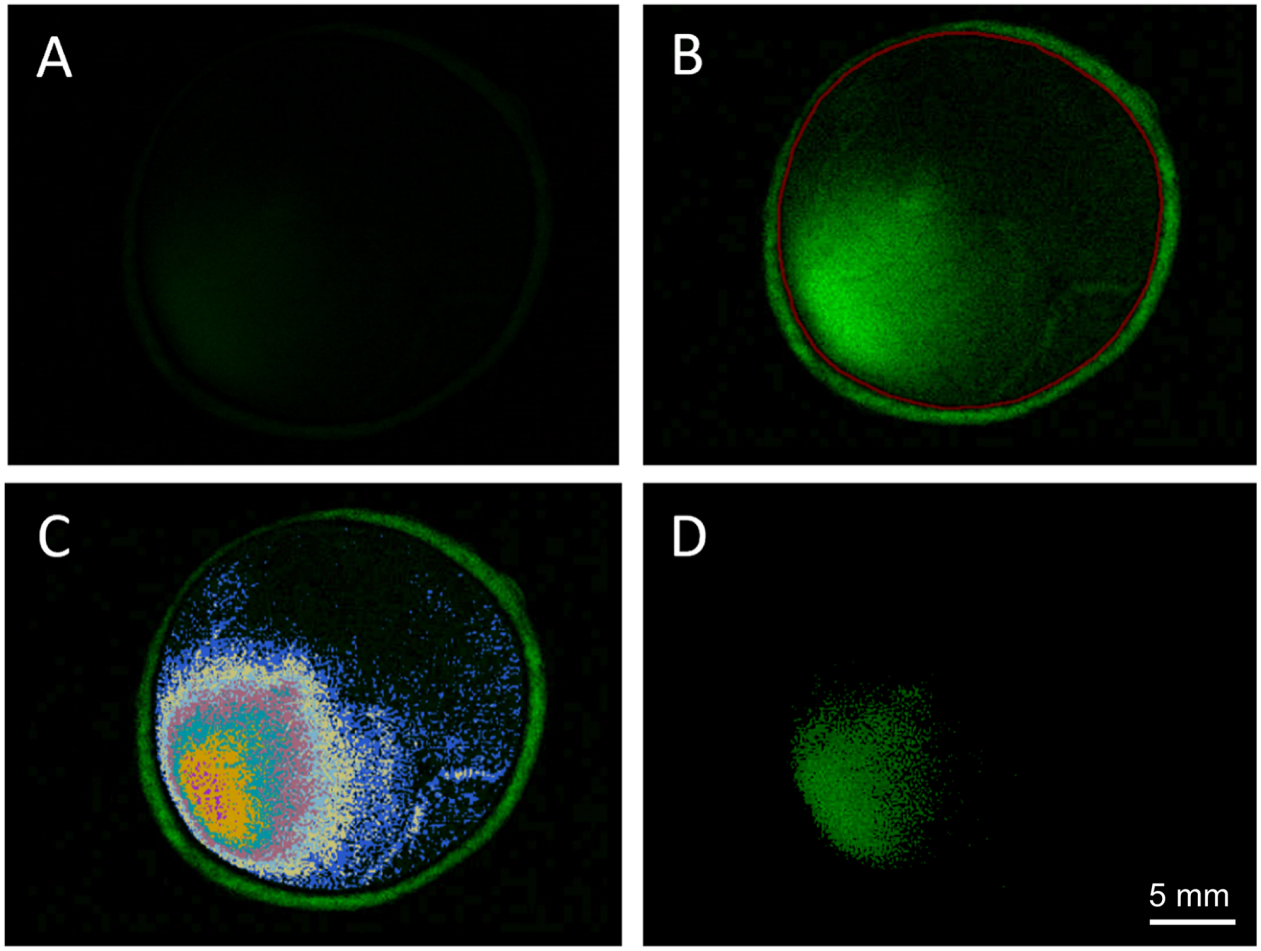
Image analysis process. (**A**) Original image. (**B**) Normalized image with *red borders*. Note that the *red line* shows the manually drawn boundary of the eyeball. (**C**) The *deep blue*, *white*, *light blue*, *pink*, *cyan*, *yellow*, and *purple regions* denote the fluorescent areas associated with threshold values of 30, 50, 70, 90, 120, 150, and 200, respectively. (**D**) The fluorescent area at a threshold of 70.

Following normalization, manual segmentation was applied to delineate the borders of each eye, facilitating analysis of precise eyeball regions (Fig. 3B). For each eye, two orthogonal diameters (nasal–temporal and inferior–superior) were measured manually, and their average was used to approximate overall eye diameter assuming circular geometry. Total eye area was calculated accordingly.

To determine the fluorescent area, threshold values (30, 50, 70, 90, 120, 150, and 200 intensity units) were systematically evaluated based on the luminance levels of the green channel in the acquired red, green, and blue (RGB) images, and the corresponding fluorescent areas were computed at each threshold setting (Fig. 3C). A threshold of 70 units was selected for all subsequent analyses (Fig. 3D), as it most closely approximated the visually apparent extent of fluorescein spread in the original images across the full sample set. The fluorescent area was defined as the contiguous region encompassing the full extent of visible fluorescence signal, including both high- and low-intensity pixels above this threshold. The percentage of fluorescent area was calculated relative to the total eye area.

To quantify dye diffusion, the maximal extent of fluorescence spread was determined for each image by measuring the largest observable diameter of the fluorescent region, with the diffusion radius defined as half of this value. In parallel, a MATLAB-based image analysis tool was used to convert normalized images to jet colormaps for visualization. An automated complementary approach was implemented using Biodock, in which the centroid of the fluorescent area was determined computationally and the radius was calculated as the distance from this centroid to the most distant boundary of the fluorescent region. To assess measurement reproducibility, this automated analysis was repeated eight times per image. Results demonstrated high consistency, with negligible variation between repeated measurements (less than 0.001 mm) and no systematic bias between manual and automated approaches, confirming the two methods yielded equivalent results.

Statistical analyses were performed using Student's *t*-test and one-way ANOVA, followed by a post hoc Tukey's honest significant difference (HSD) comparison. Significance levels were set at *P < 0.05, **P < 0.01, and ***P < 0.001. Results with no statistical significance (*P* > 0.05) are not shown in the figures.

To compare fluorescein distribution between nasal and temporal injection sites (Supplementary Fig. S1), the vitreous cavity was divided into nasal and temporal halves. Nasal areas in nasally injected eyes were compared to temporal areas in temporally injected eyes, and vice versa. The diffusion radius was evaluated relative to the predominant axis of fluorescence extension, defined as the maximal distance from the centroid of the fluorescent area to its farthest boundary. Statistical comparisons between injection sites were performed using a two-tailed Student's *t*-test.

### Fluorescein-Only Injection in In Vivo Pig Eyes

All in vivo experiments were conducted following an approved protocol of The University of Pittsburgh Institutional Animal Care and Use Committee. Three pigs (six eyes) participated in the in vivo part of the study. The parameters of the pigs included the following: miniature pig (*Sus scrofa domesticus*) with a live weight of 46 to 59 pounds (21–27 kg), an age of 6 months, and eye diameter of 18.65658 to 25.42192 mm.

In vivo pig eye injections were performed similarly as described above, exclusively administered at the temporal site, as ex vivo experiments demonstrated no significant difference between injection sites. Two types of FITC–dextran (40-kDa and 2-MDa MW) were utilized in vivo. Injections were conducted with sterile instruments under aseptic conditions after preparing the ocular surface with a 5% Betadine solution (Alcon, Geneva, Switzerland). A 30-gauge needle was used for injection. The injection site was measured 3.5 mm from the limbus to account for the smaller size of the miniature-pig eye (Fig. 2). Animals were observed for 48 hours and examined for evidence of external signs of ocular inflammation such as redness, discharge, cloudiness of the anterior segment, or abnormal posture or behavior, none of which presented. Animals were euthanized at 48 hours post-injection (hpi), and their eyes were enucleated.

Following enucleation, a corneal incision was made, and eyes were immersed in vitreous fixative for 48 hours at room temperature, with the cornea positioned upward. After fixation, eyes were rinsed with 1× PBS, bisected, and imaged as detailed above, with the only modification being that the cut was performed 8 mm from the limbus to account for the smaller size of the miniature-pig eye. Twelve images from six pig eyes were captured and analyzed.

### Fluorescein Injection in Plasmin Pretreated Ex Vivo Pig Eyes

For information about the ex vivo pig eyes, see Animals section, above. Plasmin from human plasma was obtained from Roche (10602361001; F. Hoffmann-La Roche, Basel, Switzerland) as a pre-prepared suspension of 0.5 mL (5 U), stored at 4°C, and utilized within 24 hours of receipt. Eyes received a temporal injection of 50 µL (0.5 U) of plasmin suspended in 3.2-M ammonium sulfate solution in the experimental group or of saline vehicle in the control group, followed by 2 hours of incubation before injection of fluorescein dye with 40-kDa or 2-MDa MW.^1^ Pre-injection tissue handling was described previously in the Tissue Holding and Injection Method section, except as follows: Following injection, all eyes were incubated for 2 hours at 37°C to support the enzymatic activity of plasmin.^2^ The vehicle group also underwent incubation to maintain procedural consistency. At the end of the incubation period, eyes were cooled on ice for 15 minutes. Then, 50 µL of fluorescein dye was injected at the same site (where the marker dot was placed) of the plasmin/vehicle injection and in the same manner. Subsequently, injected eyes were shaken in 1× PBS for 24 hours as described above and then fixed for 48 hours in vitreous fixative. Fixed eyes were then rinsed with PBS solution and further bisected, imaged, and analyzed as described above.

### Fluorescein-Only Injection in Ex Vivo Human Eyes

#### Human Eyes

Three non-fixed whole-globe human eyes from donors were acquired and utilized in our National Disease Research Interchange tissue repository experiments within 36 hours of tissue recovery. One eye was from a 42-year-old donor, and two eyes were from a 36-year-old. One eye from a young donor (55 years old) and an elderly donor (77 years old) were received from the Center for Organ Recovery & Education and were used in the experiment within 3 hours of recovery. The inclusion criteria for all eyes were no history of ocular disease, trauma, or surgery and no history of radiotherapy in the head area. The eye diameters ranged from 21.9114 to 25.94056 mm.

#### Experiment Outlines

The temporal injection site was 4 mm from the limbus, using surgical calipers, and marked (Fig. 2). As described above, a 30-gauge needle was employed to inject 50 µL of 40-kDa or 2-MDa dye and shaken for 24 hours. The bisection procedure, photography, and picture analysis were similar to those described earlier.

## Results

### Fluorescein Is Distributed Through Ex Vivo Pig Eyes in a Size-Dependent Manner

**Figure 4.**
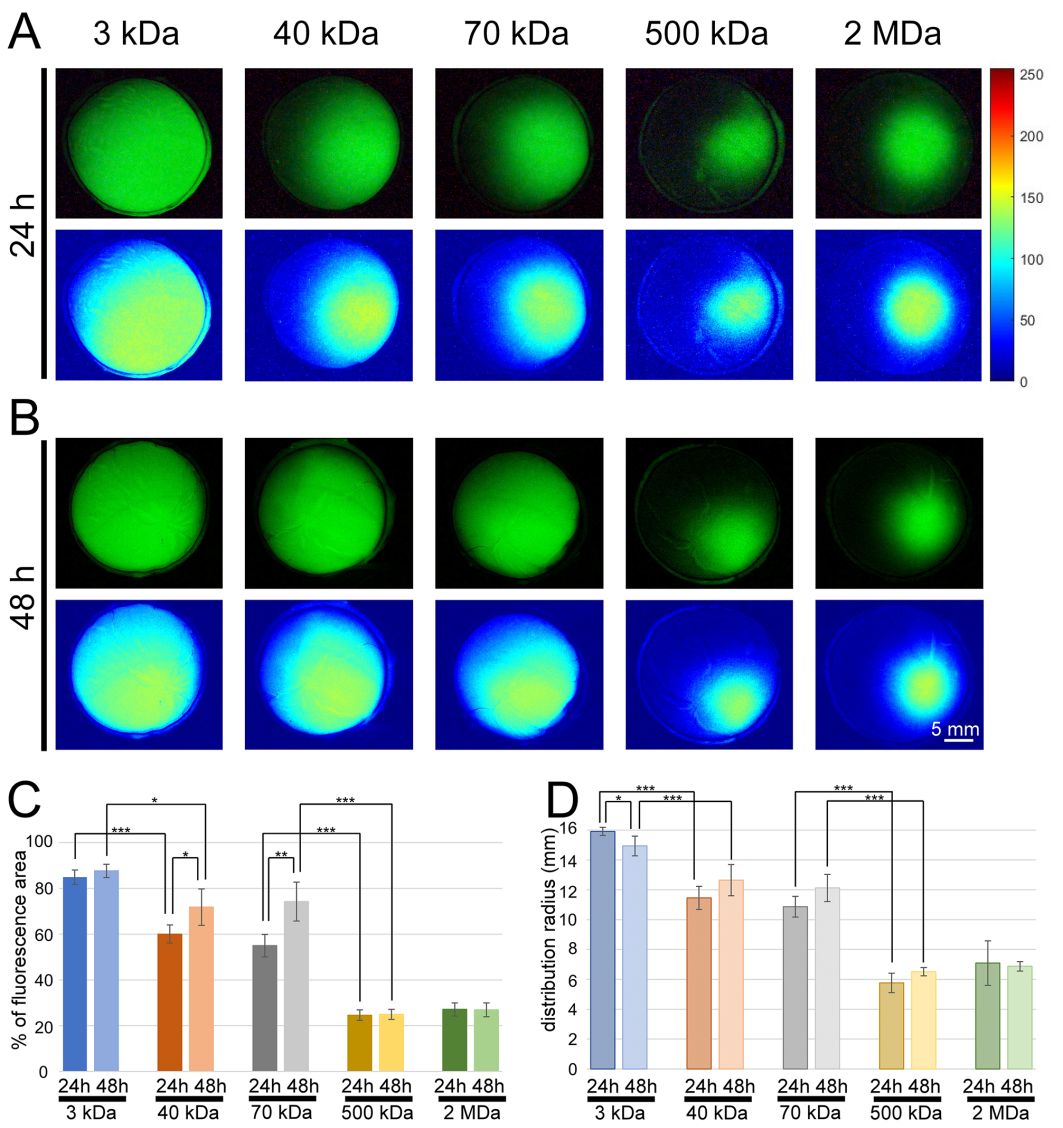
Representative fluorescence microscopic images of the intraocular distribution of anionic fluorescein dextran conjugate (FITC–dextran) dyes with five varying MWs (3 kDa, 40 kDa, 70 kDa, 500 kDa, and 2 MDa) in ex vivo pig eyes. (**A**, **B**) Images were captured at different time points: 24 hours (**A**) and 48 hours (**B**). (**C**) Bar chart of the fluorescent area relative to the total area of the eye, presented as percentage of total area. (**D**) The average distribution radius (mm) of each dye at different time points. ANOVA followed by post hoc Tukey’s HSD tests were performed, and significance levels were set at *P < 0.05, **P < 0.01, and ***P < 0.001.

Intraocular distribution of anionic fluorescein dextran dyes with five varying MWs (3 kDa, 40 kDa, 70 kDa, 500 kDa, and 2 MDa) at two different time points (24 and 48 hpi) are shown as microscopic images in Figure 4. Those images were captured and developed by fluorescence microscopy and presented after normalization using in-house software (Figs. 4A, 4B, top) and MATLAB (Figs. 4A, 4B, bottom). The fluorescent area was divided by the eye area and presented as a percentage.

The 3-kDa dyes were distributed throughout the whole eye and covered on average 84.90% ± 3.15% (*n* = 8) of the vitreous cavity by 24 hpi and 87.69% ± 2.91% (*n* = 8) by 48 hpi (Figs. 4A–C). Those results did not differ significantly, indicating that the three kDa fluorescein molecule diffuses quickly and can distribute over almost the whole pig eye after only 24 hours. The diffusion radius of the 3-kDa dye was estimated as at least 15.9 ± 0.27 mm (*n* = 8) by 24 hpi which did not significantly differ from the results obtained in the 48-hpi eyes (14.93 ± 0.66 mm; *n* = 8) (Fig. 4D). In contrast, the 40-kDa fluorescein molecule distributed over only 60.05% ± 3.95% (*n* = 8) of the cavity after 24 hours and only slightly, but significantly, more by 48 hours (71.83% ± 7.94%; *n* = 8; *P* < 0.05, Tukey's HSD), indicating that the diffusion of the 40-kDa molecule is slower than the 3-kDa molecule and seems to be impeded by the vitreous barrier (Figs. 4A–C). The average distribution radius of the 40-kDa dye was estimated as approximately 11.45 ± 0.77 mm (*n* = 8) by 24 hours, rising to 12.64 ± 1.04 mm (*n* = 8) by 48 hours (Fig. 4D); however, the results were not statistically significantly different. Significant results were also obtained for the 70-kDa dye, where the fluorescent area and distribution radius increased by the later time point; average fluorescent areas were 54.98% ± 4.87% (*n* = 8) and 74.25% ± 8.49% (*n* = 8), respectively, and average distribution radii were 10.86 ± 0.69 mm (*n* = 8) and 12.11 ± 0.91 mm (*n* = 8) at the *P* < 0.01 level (Figs. 4A–C). The fluorescent areas in eyes injected with higher MW dyes were not significantly different between the 24-hpi and 48-hpi groups (500 kDa: 24.61% ± 2.26%, *n* = 8, *P* = 0.88; 2 MDa: 27.11% ± 2.88%, *n* = 8, *P* = 0.93), and occupied much less of the cavity than the smaller MW dyes (*P* < 0.001). The average dye distribution radii in the 24-hpi groups were not significantly different from those of the respective 48 hpi groups (500 kDa: 5.76 ± 0.65 mm, *n* = 8, *P* = 0.13; 2 MDa: 7.08 ± 1.49 mm, *n* = 8, *P* = 0.83) (Fig. 4D).

Nasal and temporal injections resulted in the same outcome, and there were no indications that bursas or other vitreous structures altered the spread pattern of the injected molecules. The mean fluorescent area occupying the nasal part of nasally injected eyes did not differ from the area occupying the temporal part of temporally injected eyes at all MWs and both time points (*P* > 0.05 for all groups). We similarly found no significant difference when we compared the mean fluorescent area of the temporal part of nasally injected eyes to the nasal part of temporally injected eyes (*P* > 0.05 for all groups). In addition, we compared the distribution radii in nasally injected eyes versus temporally injected eyes at the two time points for all MWs. We found no significant differences (*P* > 0.05 for all groups).

### Higher MWs Dyes Still Have Limited Distribution in the In Vivo Pig Eyes

**Figure 5.**
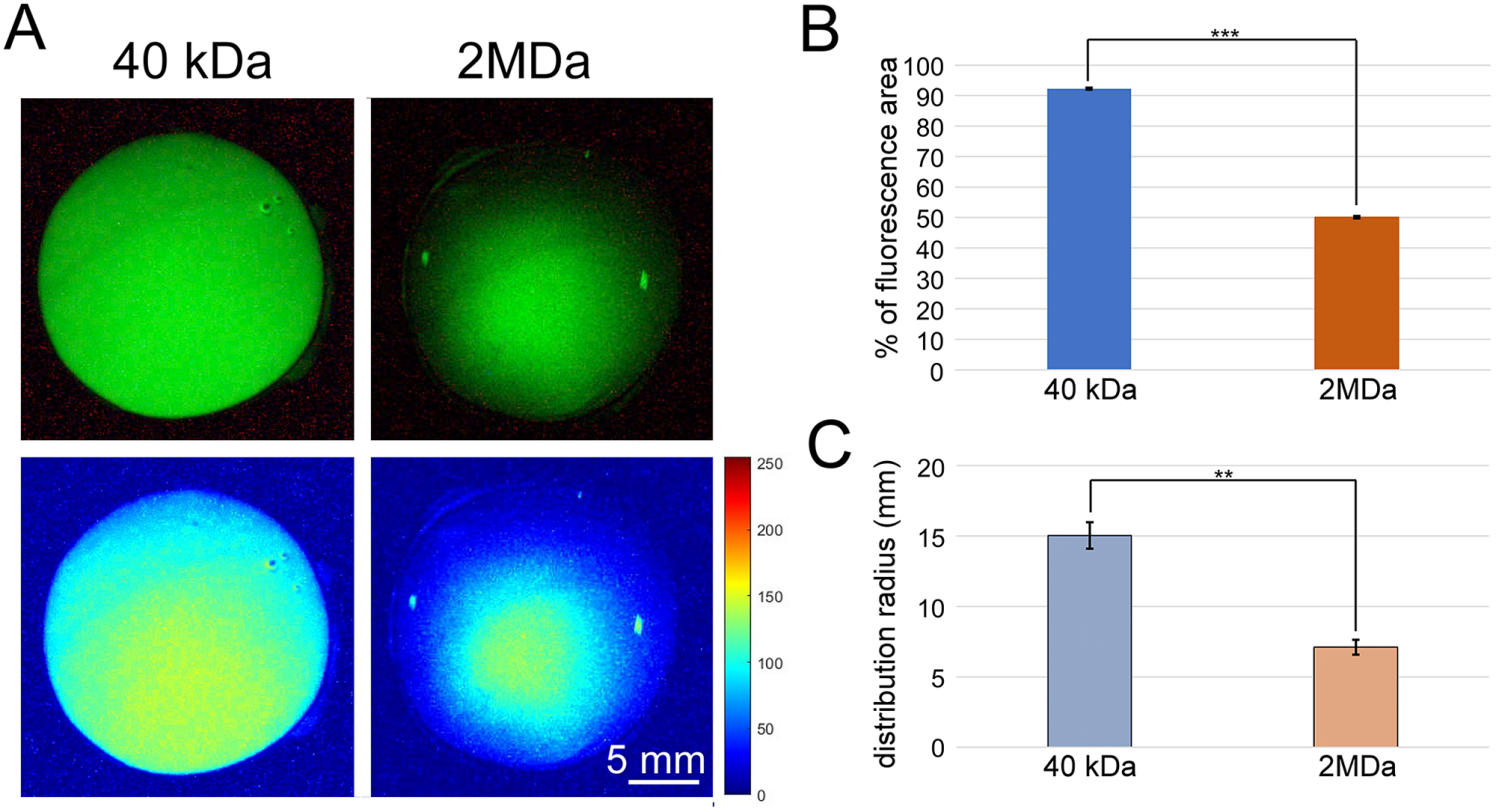
Representative fluorescence microscopic images of the intraocular distribution of anionic FITC–dextran dyes with MWs of 40 kDa and 2 MDa in the in vivo pig eyes. (**A**) Images were captured at 48 hours. (**B**) Bar chart of the fluorescent area relative to the total area of the eye, presented as percentage of total area. (**C**) The average distribution radius (mm) of each dye at different time points.

Examination of the intraocular distribution of anionic fluorescein dyes with two MWs (40 kDa and 2 MDa) in the in vivo pig eyes revealed a very different distribution pattern. Images captured by fluorescence microscope, presented as normalized (Fig. 5A, top) and after MATLAB analysis (Fig. 5A, bottom), estimated the mean fluorescence area in eyes from live animals injected with 40-kDa dye as 92.25% ± 0.30% (*n* = 3), which was significantly different from the mean distribution area of the 2-MDa dye (50.08% ± 0.38%, *n* = 3, *P* < 0.001) (Fig. 5B) at the 48-hour time point. The mean distribution radius was also significantly different between the two dyes (40 kDa: 15.04 ± 0.94 mm, *n* = 3; 2 MDa: 7.11 ± 0.53 mm, *n* = 3; *P* < 0.01) (Fig. 5C). Because the ex vivo and the in vivo experiments were performed on different pig strains and the average size of the eyes is different, the percentage of fluorescence value may not be directly compared because its value is relative to eye size. The average distribution radius is an absolute value and can be compared. However, this parameter might be slightly affected by the breed of the pigs. Interestingly, the average distribution radius of the 40-kDa dye measured in the in vivo injected eyes (15.04 ± 0.94 mm, *n* = 3) was more extensive compared to ex vivo injected eyes (12.64 ± 1.04 mm, *n* = 8). Those differences might occur because of the biologically assisted flow of fluids in a live animal’s eye. The average distribution radius of the 2-MDa dye was not significantly different between the ex vivo (6.87 ± 0.31 mm, *n* = 8) and the in vivo (7.11 ± 0.53 mm, *n* = 3) experiments, indicating that the assisted fluids flow may have a limit and that large molecules are impeded in the live animal, possibly by vitreous structures.

### Plasmin Pretreatment Facilitates Medium- But Not Large-Size Dye Distribution in Ex Vivo Pig Eyes

**Figure 6.**
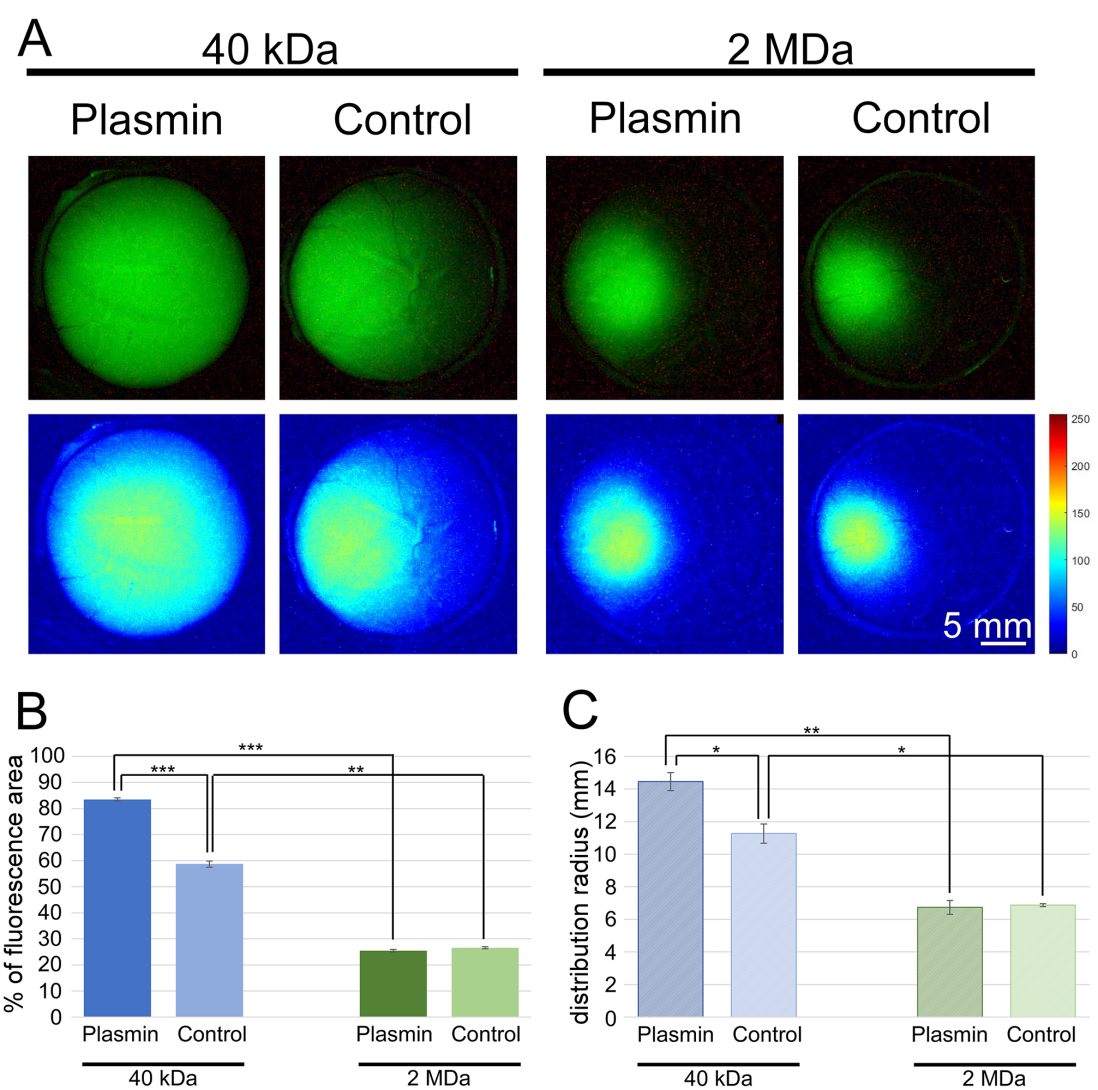
Fluorescence microscopic images of the intraocular distribution of anionic 40-kDa and 2-MDa MW FITC–dextran dyes in ex vivo pig eyes, initially treated with plasmin. (**A**) Images presented at a threshold of 70 (*top*) and images developed by MATLAB (*bottom*) were captured after incubation of 24 hours. (**B**) A bar chart of the fluorescent area out of the whole eye area is presented as percentages. (**C**) The average distribution radius (mm) of each dye at different time points. ANOVA followed by post hoc Tukey’s HSD tests were performed, and significance levels were set at *P < 0.05, **P < 0.01, and ***P < 0.001.

Images of ex vivo pig eyes injected with plasmin or the saline vehicle before the fluorescein injection are presented in Figure 6A. The average percentage of fluorescence area in control eyes injected with saline (40 kDa: 58.70% ± 1.15%, *n* = 2; 2 MDa: 26.60% ± 0.39%, *n* = 2) matched the ex vivo experiments of the same MWs (40 kDa: 60.05 ± 3.9 mm, *n* = 8, *P* = 0.81; 2 MDa: 27.11 ± 2.88 mm, *n* = 8, *P* = 0.9). No significant differences were observed between the average radius of distribution in vehicle eyes (40 kDa: 11.26 ± 0.58 mm, *n* = 2; 2 MDa: 6.87 ± 0.08 mm, *n* = 2) compared to ex vivo injected eyes from the first experiment, which were not exposed to heating (40 kDa: 11.45 ± 0.77 mm, *n* = 8, *P* = 0.86; 2 MDa: 7.08 ± 1.49 mm, *n* = 8, *P* = 0.91). This indicates that injection of saline and/or heating the eye for 2 hours does not materially affect the fluorescein spread. The average percentage of fluorescence area in eyes injected with plasmin before 40-kDa dye injection significantly increased (83.50 ± 0.64 mm, *n* = 3, *P* < 0.001) (Fig. 6B). It almost reached the percentage measured for untreated eyes injected with 3-kDa dye from the first experiment (84.90 ± 3.15 mm, *n* = 3), indicating that plasmin treatment affects the vitreous structure and can improve the diffusion of molecules with MWs of at least up to 40 kDa. No significant difference was identified between plasmin (fluorescence area = 25.42% ± 0.47%; radius = 6.73 ± 0.42 mm; *n* = 3) and saline-treated eyes (fluorescence area = 26.60% ± 0.39%; radius = 6.87 ± 0.08 mm; *n* = 2) before 2-MDa dye injection, indicating that plasmin is not able to dissolve the whole vitreous barrier during the 2 hours of incubation, as vitreous structures still impede large molecules such as 2-MDa dye. Measurements of the distribution radius showed similar results (Fig. 6C). The average distribution radius of 40-kDa dye in eyes injected with saline (11.26 ± 0.58 mm) was significantly more restricted than the average radius measured in eyes treated with plasmin (14.45 ± 0.54 mm; *P* < 0.05). No significant difference in the radius of fluorescence was identified in eyes injected with 2-MDa dye. These results support the previous assessment that vitreolysis using plasmin is inefficient and cannot improve the distribution of large molecules with MWs of at least 2 MDa.

### The Mid-Size Dye Distributed Farther in Ex Vivo Human Eyes Than the Average Seen in Ex Vivo Pigs. The Largest MW Dye Was Similarly Restricted but With a Different Diffusion Pattern

**Figure 7.**
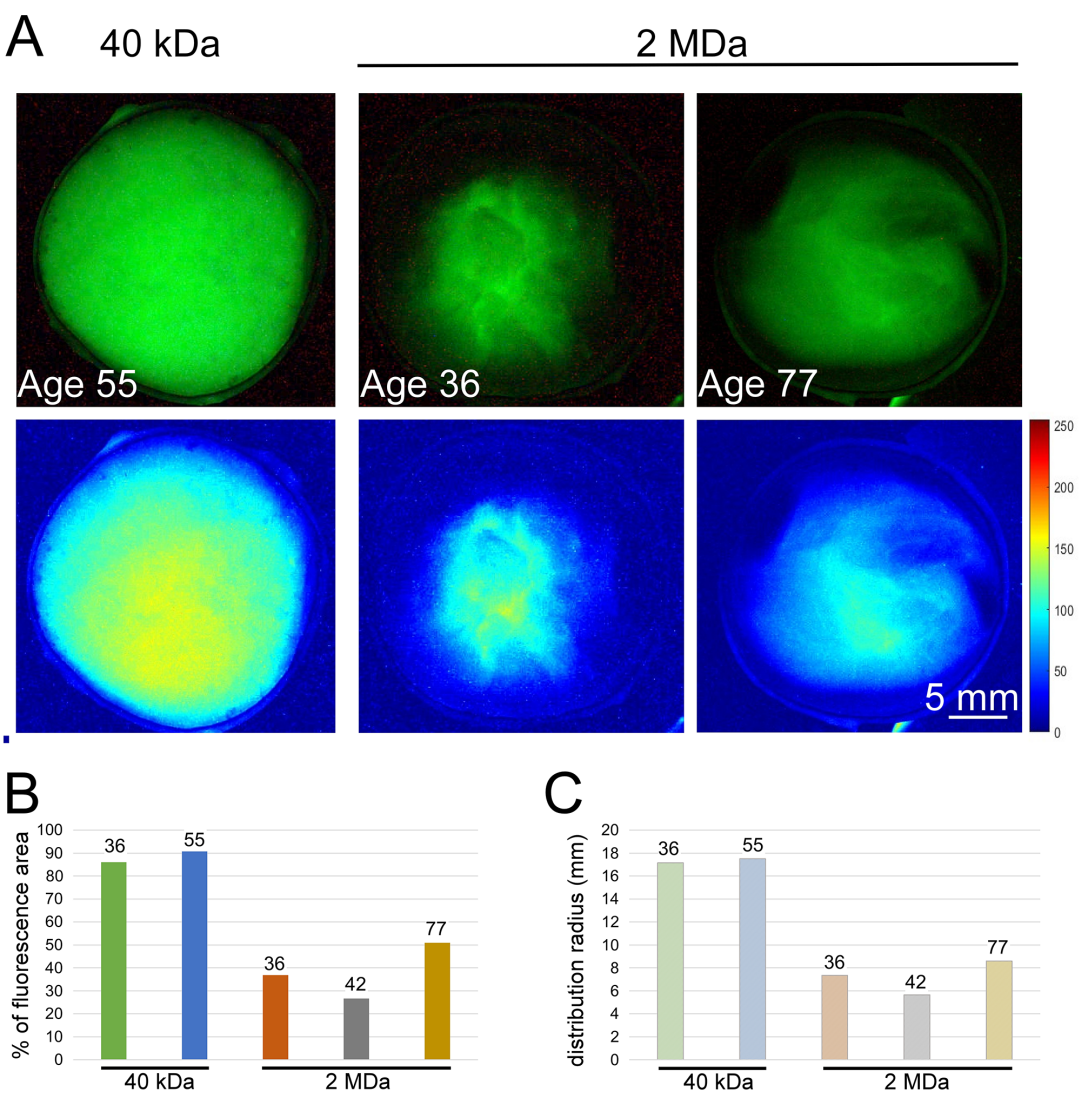
Fluorescence microscopic images of the intraocular distribution for 40-kDa and 2-MDa MW FITC–dextran dyes in ex vivo human eyes. (**A**) Images presented at a threshold of 70 (*top*) and images developed by MATLAB (*bottom*) were captured after 24 hours of incubation. (**B**) A bar chart of the fluorescent area out of the whole eye area is presented as percentages. The ages of the donors are given above each bar. (**C**) The average distribution radius (mm) of each dye at different time points.

Intraocular injections of anionic fluorescein dyes with two MWs (40 kDa and 2 MDa) into ex vivo human eyes yielded variable results. The distribution of 40-kDa fluorescein was approximately homogeneous through the two human eyes examined, with average percentage fluorescent areas of roughly 86% and 90.7% (in eye donors ages 36 and 55 years, respectively) (Figs. 7A, 7B). The radii of distribution measured in those eyes were also quite similar (17.14 and 17.51 mm, respectively) (Fig. 7C). The distribution of 2-MDa fluorescein was less homogeneous, more variable in its pattern and values between the eyes, and more limited in spread compared with the 40-kDa dye (Fig. 7A). The fluorescent areas measured in human ex vivo eyes from donors at 36, 42, and 77 years were 36.82, 26.67, and 50.89 mm, respectively (Fig. 7B). The distribution radii measured for the 2-MDa molecules were smaller than those of the 40-kDa dye and ranged from 5.65 to 8.59 mm (Fig. 7C). Interestingly, the oldest donor (age 77 years) observed the widest distribution of the heaviest molecule in the eye. That eye was mentioned as a myopic eye in the donor's medical records.

## Discussion

In our initial experiments in ex vivo pig eyes, we investigated the distribution of anionic FITC–dextran dyes of five different MWs (3 kDa, 40 kDa, 70 kDa, 500 kDa, and 2 MDa), the sizes of which correlate with their MWs. In these experiments, we found that larger MW and size fluorescein molecules displayed a smaller distribution radius and fluorescence area. These findings suggest that MW and size matter greatly when using an intravitreal route of injection, which is critical information when developing a new drug or delivery system. Notably, dyes with similar MWs (such as 40 kDa vs. 70 kDa and 500 kDa vs. 2 MDa) exhibited identical distribution patterns. Dyes with a 3-kDa showed complete distribution in the vitreous cavity by 24 hours, whereas those with slightly larger weights (40 kDa and 70 kDa) tended to continue distribution beyond 24 hpi. Conversely, the highest MW dyes (500 kDa and 2 MDa) showed a limited and consistent pattern of spread across both time points, indicating that the vitreous barrier likely influences and limits the spread of higher MW drugs throughout different time points.

Furthermore, although we found no difference in dye distribution between temporal and nasal injections, dyes with a MW higher than 3-kDa showed spread limited to the side of the injection. This observation suggests that, in a clinical setting, the site of injection may not significantly influence drug distribution. However, when treating a specific pathology at a particular site using drugs with higher MWs, injecting the drug directly at or immediately adjacent to that site should be considered.

As a next step, we aimed to investigate whether processes occurring in the living eye could contribute to and potentially influence dye distribution. To achieve this, we utilized the in vivo pig model. We chose to perform injections exclusively at the temporal site for these experiments, as earlier ex vivo experiments showed no significant difference between injection sites. Additionally, we decided to examine only the 40-kDa and 2-MDa MWs for several reasons. First, although the 3-kDa dye resembles the MW of vancomycin (1.45 kDa), a tricyclic glycopeptide antibiotic commonly used together with ceftazidime, a third-generation cephalosporin, to treat bacterial endophthalmitis via the intravitreal route, the 3-kDa dye already exhibited a full spread pattern in ex vivo experiments.^10^ Therefore, we did not find further interest in studying it in vivo. Second, similar behavior was observed between the 40-kDa and 70-kDa and between the 500-kDa and 2-MDa molecule sets. The distribution kinetics of the 40-kDa and 2-MDa molecules are particularly interesting as they hold significant clinical and research relevance. The 40-kDa weight is close to that of the anti–vascular endothelial growth factor (VEGF) molecule ranibizumab, commonly used in clinical practice to treat multiple ocular conditions such as neovascular age-related macular degeneration, macular edema of different etiologies, and choroidal neovascularization. In comparison, the 2-MDa weight resembles that of adeno-associated virus (AAV) in molecular size, a common gene therapy vector.^11^^,^^12^ Our study revealed different distribution kinetics for dyes with lower MWs and smaller sizes in ex vivo pig eyes compared to in vivo. Although the ex vivo model exhibited partial diffusion of a dye with a MW of 40 kDa at 48 hpi, the in vivo pig model demonstrated almost complete diffusion of that dye throughout the vitreous cavity simultaneously. It seems that living body factors (hypothetically, such as body temperature, blood and aqueous flow presence, and other physical and chemical reactions inherent to the living body) may influence the spread of lower MW dyes, presumably of lower MW treatment molecules in the clinic. Interestingly, in the living pig eye, the 2-MDa dye showed only the same partial spread restricted close to the injection site observed in the earlier ex vivo experiments. This is probably due to physical barriers in both models that limit the spread of high-MW and molecular-size drugs. These observations underscore the critical importance of selecting the appropriate research model when studying the barrier function of the vitreous and when testing the spread of future candidate drugs throughout the eye.

It should also be noted that the ex vivo and in vivo porcine experiments were conducted using different pig breeds: Yorkshire, Duroc, and Hampshire pigs for the ex vivo experiments and miniature pigs for the in vivo experiments. These breeds differ not only in body and eye size but potentially also in vitreous composition and structural organization. The domestic pig breeds used in the ex vivo experiments have larger eyes and vitreous volume, whereas the miniature pigs have smaller eyes with correspondingly reduced vitreous volumes. Differences in eye size among breeds may be accompanied by differences in the relative concentrations of collagen and hyaluronic acid, the degree of vitreous gel organization, and the viscoelastic properties of the vitreous body, all of which could independently influence intravitreal dye distribution. Although the consistency of size-dependent diffusion patterns observed across both breeds lends confidence to the generalizability of our findings, the potential contribution of interbreed differences in vitreous composition to the observed differences in absolute distribution radius between ex vivo and in vivo experiments cannot be fully excluded and warrants consideration in future comparative studies.

The next question in our study was whether disturbing vitreous integrity would improve dye distribution. Pars plana vitrectomy and pharmacological vitreolysis are two main approaches that allow interruption of vitreous structure. Among them, pharmacological vitreolysis is less invasive. It has primarily been utilized in clinical practice to induce posterior vitreous detachment and reduce abnormal vitreomacular traction to protect the retina (or “intraocular structures”) from physical strain.^13^^,^^14^

The process of pharmacological vitreolysis involves the intravitreal administration of one or more of various proteolytic enzymes, which liquefy the vitreous and/or detach adhesions between the posterior hyaloid and the internal limiting membrane.^13^^,^^14^ Gad Elkareem et al.^2^ performed pharmacological vitreolysis in ex vivo pig eyes using a 0.5-IU intravitreal injection of plasmin followed by incubation at 37°C for 2 hours.^2^ We adopted a similar technique in our experiments and observed that pretreatment with plasmin enhanced the distribution of a lower MW (40 kDa) dye compared to a higher MW (2 MDa) one. The exact reason for this phenomenon is not entirely apparent. Gad Elkareem et al.^2^ reported that plasmin primarily digests proteins with a MW above 50 kDa. Our observations may be related to the incomplete disruption of vitreous fibers larger than that threshold by plasmin and the persistent blocking effect those fibers exert on more prominent size and larger MW particles. Importantly, human plasmin has a MW of ∼85 kDa, which means the enzyme itself may have been restricted in its movement and could only disrupt vitreous structure as it slowly moved through it. It is possible that had we either increased our incubation time or included a group of eyes that had plasmin injected in the pole *opposite* the dye injection that the distribution pattern would have changed to show a greater spread. The limited spread observed may have potentially important clinical and research implications, as it could indicate that pharmacological vitreolysis may be insufficient to allow full distribution of drugs in cases where high MW and size substances are used, such as AAV. Consequently, this observation warrants further investigation in an in vivo animal model, as the physiological conditions of the living eye may substantially alter the outcome. Our in vivo porcine experiments demonstrated that living physiological factors might be a contributing factor in enhancing the distribution of the 40-kDa dye relative to ex vivo conditions. It is therefore plausible that plasmin itself, being a molecule of approximately 85 kDa, would distribute more extensively within a living vitreous than in an ex vivo setting, potentially enabling broader enzymatic disruption of the vitreous scaffold and more complete vitreolysis than was observed here. This could in turn facilitate the distribution of subsequently injected molecules of higher MW, including those in the 2-MDa range. Future studies should therefore evaluate plasmin and alternative pharmacological vitreolysis agents in in vivo models to determine whether the in vivo physiological environment meaningfully improves their efficacy. It should also be noted that the plasmin used in the present experiments was human plasmin obtained from a research-grade supplier and has not been approved for intravitreal use in human patients. Ocriplasmin, a truncated recombinant form of plasmin that was previously the only pharmacological vitreolysis agent to receive clinical approval for treatment of vitreomacular adhesion and traction, was withdrawn from the market and is no longer commercially available.^15^^,^^16^ Pharmacological vitreolysis therefore currently has no approved agent in clinical use, underscoring the need for continued research into safe and effective enzymatic approaches to vitreous modulation, including their potential role in improving intravitreal drug delivery.

**Figure 8.**
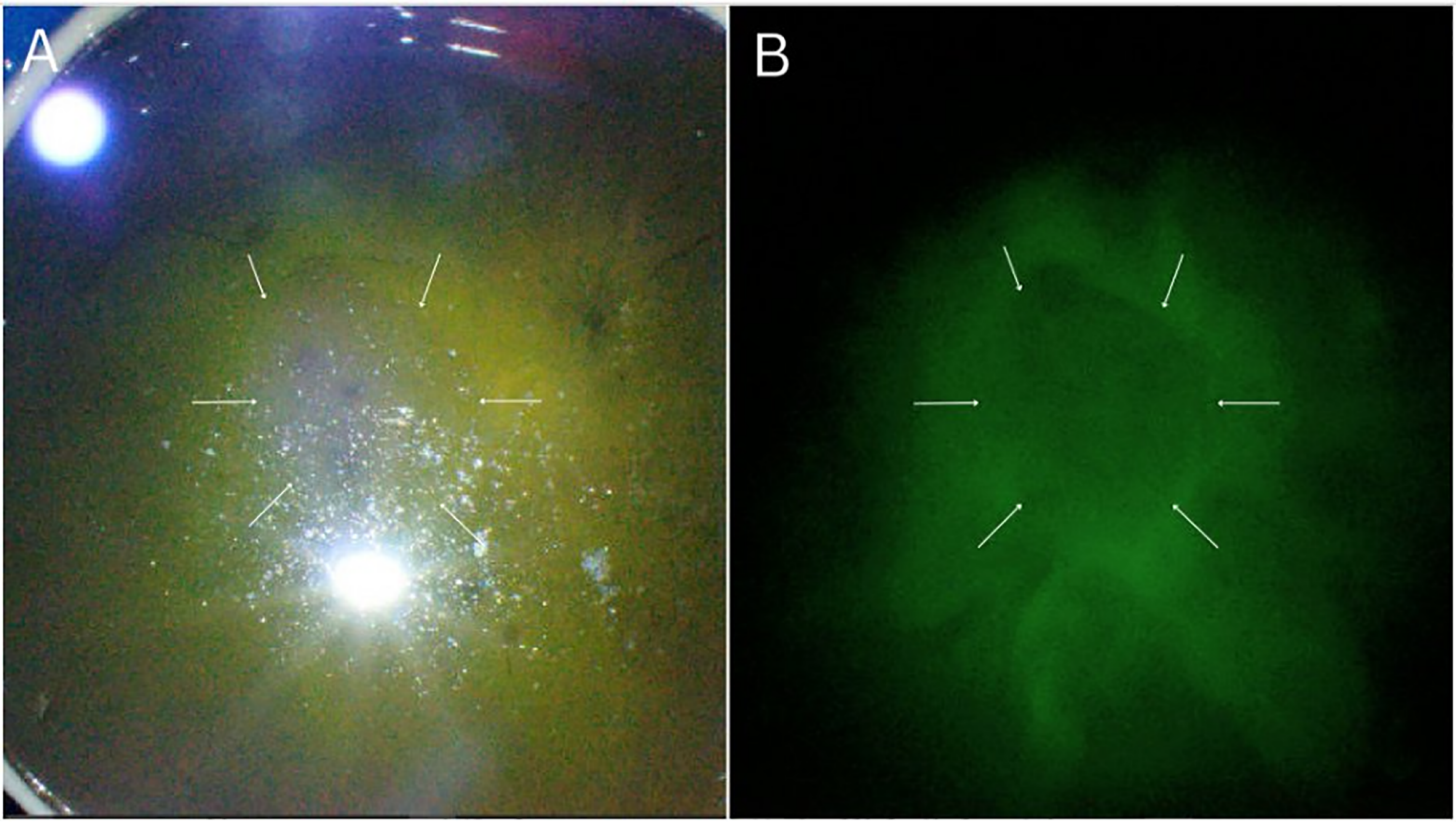
(**A**, **B**) Multicolor (**A**) and fluorescence (**B**) images of a 36-year-old female donor eye 24 hours after injection with 2-MDa MW FITC–dextran dye. Note the blockage of fluorescein distribution over the macular area (*arrows*) where a prominent macular bursa is present.

The final step of our study involved implementing all observations from previous experiments into the ex vivo human eye model. The young ex vivo human eyes exhibited dye spread patterns similar to those we observed in the in vivo pig eyes: the 40-kDa dye showed almost complete diffusion, and the 2-MDa dye had severely limited spread. Notably, the distribution shape in the human eye revealed prominent vitreous bursas not observed in the pig eye. A macular bursa in the human eye appeared to interrupt the dye distribution over the macular area, resulting in inconsistent and limited distribution (Fig. 8). Therefore, the presence of the bursa should be considered when treating macular pathologies with high-MW drugs. On the one hand, the macular bursa may impede the diffusion of the drug over the macular area, potentially reducing treatment effectiveness. On the other hand, injecting the high-MW drug into the macular bursa could transform it into a reservoir for slow drug release, concentrating the drug over the macular area and enhancing its effectiveness. Some studies in gene therapy have already demonstrated that direct pre-retinal administration of AAV in animal models improves the efficiency of viral transduction.^17^ Our findings may support this and suggest changing injection strategies when targeting the macular area rather than abandoning studies whose initial results reveal low transduction.

The prominence of the macular bursa in human eyes and its apparent absence in pig eyes reflect a broader and clinically important difference in vitreous microarchitecture between the two species that extends beyond bursae alone. The human vitreous harbors an interconnected cisternal system of considerable complexity, first characterized by Jongebloed and Worst^18^ using ink injection techniques, that is comprised of the bursa premacularis, the area of Martegiani overlying the optic disc, Cloquet's canal, prevascular vitreous fissures, and a surrounding circle of cisterns—all representing liquid-filled compartments of lower resistance embedded within the formed vitreous gel.^1^ These structures are nearly universal in adult human eyes and have been characterized in vivo using swept-source optical coherence tomography (OCT), and their topographic relationships have been shown to evolve with age.^19^ The porcine vitreous, although broadly comparable to the human vitreous in overall size and mechanical properties, does not appear to harbor an equivalent cisternal system of similar complexity, and rheological similarity between species does not necessarily predict equivalent drug diffusion behavior. Shafaie et al.^20^ demonstrated that, despite similar viscoelastic properties, porcine vitreous exhibits significantly lower steady-state flux and diffusion coefficients for fluorescein compared to human vitreous, suggesting that structural and compositional differences at the level of the collagen–hyaluronate network contribute independently to the diffusion disparities observed between species. Although the conclusion of that study—that animal vitreous models are poor surrogates for human vitreous with respect to fluorescein diffusion kinetics—may appear to challenge the use of porcine eyes in the present work, it is important to distinguish between two fundamentally different experimental questions. Shafaie et al.^20^ quantified the diffusion coefficient and steady-state flux of a single small molecule across extracted vitreous of different species, a question that is highly sensitive to subtle differences in vitreous composition and matrix density. Our study, by contrast, examined size-dependent spatial distribution patterns of molecules spanning three orders of magnitude in MW within intact whole globes, a question governed primarily by the gross barrier properties and macroarchitectural organization of the vitreous rather than by species-specific differences in matrix diffusivity. The porcine model therefore remains a valid and informative experimental system for studying size-dependent vitreous barrier function, even while acknowledging that it does not fully replicate the quantitative diffusion parameters of human vitreous, and the differences in dye distribution patterns between pig and human eyes may reflect a combination of microarchitectural complexity, cisternal organization, and intrinsic matrix composition, all of which should be considered when extrapolating findings from porcine models to the human clinical context. Future studies should investigate how these structural differences specifically influence the distribution of intravitreal agents of varying molecular sizes.

Age-related changes in vitreous architecture likely contribute to the differences observed in our human samples. With aging, the vitreous undergoes liquefaction, syneresis, and eventual posterior vitreous detachment (PVD), processes that alter or diminish cisternal structures such as the premacular bursa. Consistent with this, the older donor eye in our study demonstrated greater distribution of the 2-MDa dye and lacked a prominent bursa. This may reflect vitreous syneresis or PVD, both of which reduce structural barriers and facilitate the mobility of larger molecules. Notably, the donor record identified this eye as myopic, which may represent an additional contributing factor beyond age alone. Myopia is associated with accelerated vitreous liquefaction independent of the aging process: In myopic eyes, vitreous syneresis occurs earlier, is more extensive, and increases with the degree of myopia, driven by molecular changes that promote dissociation of hyaluronic acid from collagen and the formation of liquefied lacunae within the gel matrix, thereby reducing structural barriers to the diffusion of large molecules.^21^^,^^22^ The wider distribution of the 2-MDa dye in this donor therefore likely reflects the combined effect of advanced age and myopia, both of which independently promote vitreous degeneration and may synergistically reduce the barrier properties of the vitreous. Patient age and refractive status may therefore be clinically relevant variables to assess prior to intravitreal treatment, and preoperative imaging of the vitreous structure could provide valuable information about the degree to which a given patient’s vitreous is likely to impede or facilitate drug distribution.

However, these biologically driven interpretations must be considered alongside methodological limitations inherent to the use of ex vivo human tissue. In the present study, the time elapsed between tissue recovery and fluorescein injection varied considerably across donors: eyes from the 36- and 42-year-old donors were utilized within 36 hours of recovery, whereas those from the 55- and 77-year-old donors were used within 3 hours of recovery. This variability may have introduced differences in vitreous structural integrity between donor groups that could independently influence the dye distribution patterns observed in Figure 7. Additionally, PBS was used as the incubation medium during the 24-hour dye diffusion period rather than a supportive or oxygenated solution, a factor that may have accelerated postmortem alterations in vitreous rheological properties. The vitreous humor is among the most resilient postmortem tissues due to its avascular and isolated nature, and its composition is preserved longer than other body fluids due to the limited permeability of the blood–retinal barrier; however, progressive changes in the collagen–hyaluronate scaffold do occur with time after death.^23^ Studies of postmortem porcine vitreous have demonstrated that 24 hours of storage at 4°C produces detectable reductions in type II collagen staining intensity, although without the formation of new breakdown products, indicating relative but not complete structural preservation over this period.^23^^,^^24^ Internal evidence from our porcine experiments argues against significant postmortem tissue degradation as the primary determinant of the size-dependent diffusion patterns reported here. Ex vivo porcine eyes incubated for up to 48 hours in PBS, conditions involving considerable postmortem tissue exposure, yielded size-dependent distribution patterns consistent in their overall character with those observed in the in vivo pig eyes examined at 48 hours post-injection under fully maintained physiological conditions. Most critically, the 2-MDa dye exhibited restricted, localized distribution near the injection site in both settings, regardless of whether the vitreous was in a living or postmortem state, providing the strongest evidence that this restricted distribution reflects a genuine structural barrier property of the vitreous rather than a postmortem artifact. Although the 40-kDa dye did show somewhat wider distribution in in vivo compared to ex vivo eyes, consistent with the contribution of living physiological factors discussed earlier, this difference does not undermine the conclusion that the fundamental size-dependent pattern of distribution is preserved across both conditions. This line of reasoning cannot, however, be directly extrapolated to the human eye experiments, where inter-donor variability in postmortem interval, extended time from tissue recovery to injection in some donors, and the use of non-oxygenated incubation media represent confounding factors that cannot be fully excluded. Future studies using human donor eyes should aim to standardize the tissue recovery-to-experiment interval, utilize oxygenated and metabolically supportive incubation media, and incorporate vitreous imaging modalities, such as those described by Sebag,^25^ to verify structural integrity prior to experimentation and identify any postmortem architectural changes that may confound the interpretation of intravitreal diffusion data.

We believe our results on the distribution patterns of FITC–dextran molecules differ from those described in the study by Peeters et al.^3^ for several reasons. First, bovine vitreous may behave differently compared to the models used in our study. Second, our study was conducted on the whole vitreous in a closed-eye system, demonstrating that the vitreous may have a multilayer meshwork structure that influences drug distribution. Although a part of the vitreous may not show blockage in the distribution even for high-molecular-weight dyes such as 2 MDa (with a molecular radius of 27 nm), blockage in distribution can be observed when all layers of the whole vitreous are present.^4^ This indicates that the average mesh size of the pig and human vitreous could be much smaller than the 550 ± 50 nm in the previous study conducted on bovine vitreous.^4^

We investigated the distribution patterns of FITC–dextran molecules as surrogates for a range of therapeutically relevant intravitreal agents: the smaller MW dextrans (3 kDa) resemble the size of commonly used intravitreal antibiotics, the mid-range dextrans (40 kDa) approximate the MW of anti-VEGF agents, and the largest dextrans (2 MDa) are comparable in size to AAV vectors used in ocular gene therapy. Like these therapeutic agents, FITC–dextrans are hydrophilic molecules, making molecular size a key shared parameter governing their intravitreal distribution. The use of FITC–dextrans across a standardized range of MWs from a single supplier was a deliberate methodological choice, designed to isolate the effect of molecular size on vitreous barrier function while minimizing confounding variables. It is important to acknowledge, however, that dextrans do not fully replicate the physicochemical complexity of these therapeutic agents, as intravitreal drugs vary considerably in surface charge, molecular conformation, structural flexibility, and binding interactions with vitreous components such as collagen fibers and glycosaminoglycans—properties that may independently influence intravitreal distribution beyond molecular size alone. Our findings therefore provide a framework for understanding size-dependent diffusion constraints within the vitreous but should be interpreted with the caveat that these additional physicochemical parameters may further modulate the distribution of specific therapeutic agents. Understanding these structure–distribution relationships is essential for optimizing future intravitreal therapeutics.

## Supplementary Material

Supplement 1

Supplement 2
