# Supplementary material for: Comparative Analysis of Intravitreal Diffusion Patterns Across Ex Vivo Human and In Vivo/Ex Vivo Animal Models
Source: Invest Ophthalmol Vis Sci. 2026 May 20;67(5):56. doi: 10.1167/iovs.67.5.56 (PMC13206738; doi:10.1167/iovs.67.5.56)
Supplement: Supplement 1 [file iovs-67-5-56_s001.pdf]

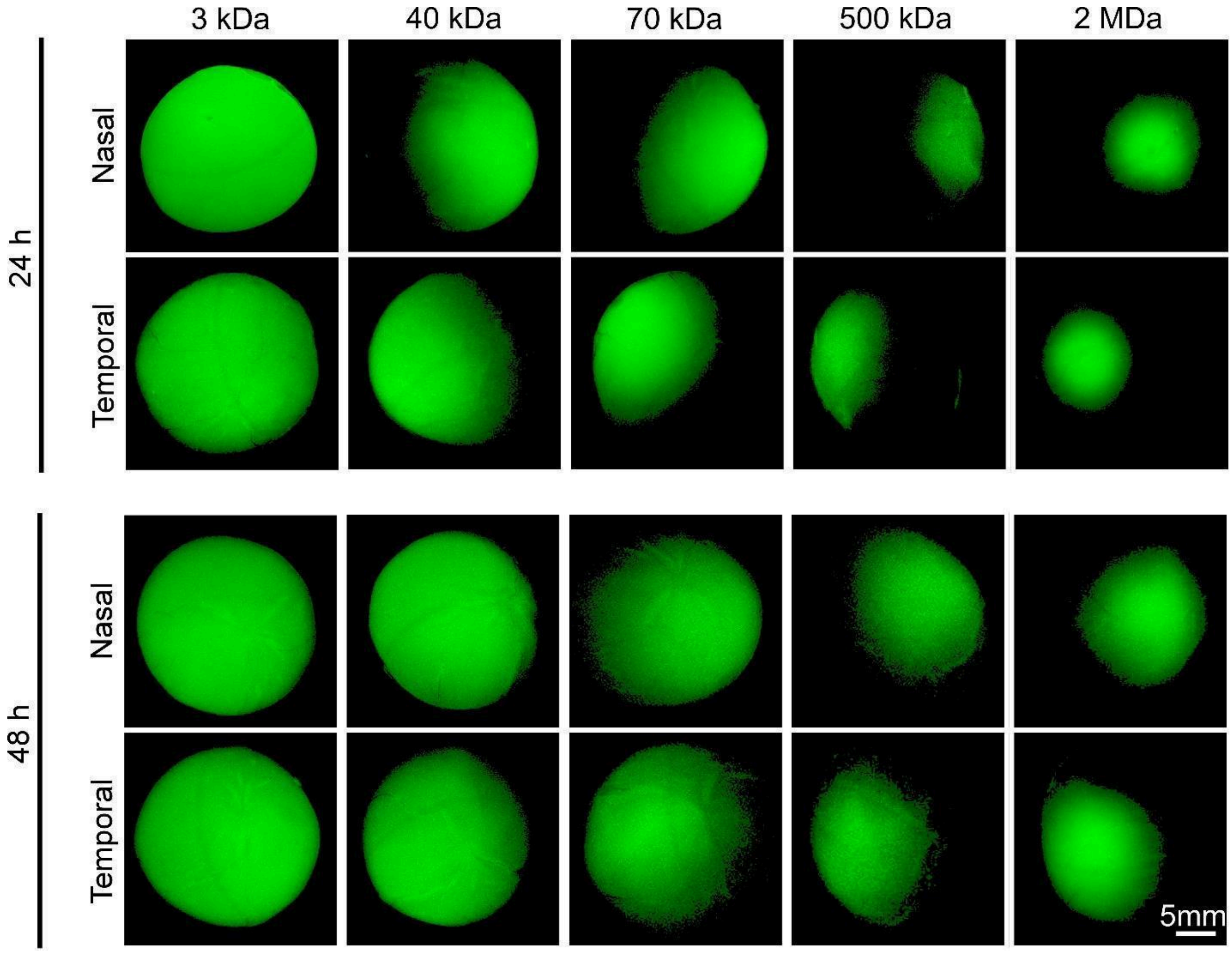


**Supp Figure S1:** Directional analysis of fluorescein distribution following nasal versus temporal intravitreal injections in *ex-vivo* pig eyes. Representative fluorescence images of *ex-vivo* pig eyes injected with FITC-dextran of increasing molecular weights (3 kDa, 40 kDa, 70 kDa, 500 kDa, and 2 MDa), shown at 24 and 48 hours post-injection. For each molecular weight and time point, paired eyes injected from nasal and temporal sites are presented. Fluorescence distribution patterns were visually assessed for directional bias relative to the injection site. While local asymmetry in fluorescence spread was observed, particularly at higher molecular weights, no consistent differences in overall distribution or diffusion extent were detected between nasal and temporal injection sites.
