# Supplementary material for: Comparative Analysis of Intravitreal Diffusion Patterns Across Ex Vivo Human and In Vivo/Ex Vivo Animal Models
Source: Invest Ophthalmol Vis Sci. 2026 May 20;67(5):56. doi: 10.1167/iovs.67.5.56 (PMC13206738; doi:10.1167/iovs.67.5.56)
Supplement: Supplement 2 [file iovs-67-5-56_s002.pdf]

Supp Table S1: Eye dimensions

Model	N (eyes)	Mean diameter (mm)	Range (mm)
Ex-vivo pig	90 (80+10)	22.77385	19.93514 - 25.62603
In-vivo mini pig	6	20.33165	18.65658 - 25.42192
Human	5	23.50447	21.9114 - 25.94056
